# Iron homeostasis in neuronal cells: a role for IREG1

**DOI:** 10.1186/1471-2202-6-3

**Published:** 2005-01-24

**Authors:** Pabla Aguirre, Natalia Mena, Victoria Tapia, Miguel Arredondo, Marco T Núñez

**Affiliations:** 1 Department of Biology, Faculty of Sciences, University of Chile, Santiago, Chile; 2 Millennium Institute for Advanced Studies in Cell Biology and Biotechnology, Santiago, Chile; 3 Micronutrients Unit, Instituto de Nutrición y Tecnología de los Alimentos, University of Chile, Santiago, Chile

## Abstract

**Background:**

Iron is necessary for neuronal function but in excess generates neurodegeneration. Although most of the components of the iron homeostasis machinery have been described in neurons, little is known about the particulars of their iron homeostasis. In this work we characterized the response of SH-SY5Y neuroblastoma cells and hippocampal neurons to a model of progressive iron accumulation.

**Results:**

We found that iron accumulation killed a large proportion of cells, but a sub-population became resistant to iron. The surviving cells evoked an adaptative response consisting of increased synthesis of the iron-storage protein ferritin and the iron export transporter IREG1, and decreased synthesis of the iron import transporter DMT1. Increased expression of IREG1 was further substantiated by immunocytochemistry and iron efflux experiments. IREG1 expression directly correlated with iron content in SH-SY5Y and hippocampal cells. Similarly, a high correlation was found between IREG1 expression and the rate of iron efflux from SH-SY5Y cells.

**Conclusions:**

Neuronal survival of iron accumulation associates with increased expression of the efflux transporter IREG1. Thus, the capacity of neurons to express IREG1 may be one of the clues to iron accumulation survival.

## Background

Because of its intense oxidative metabolism, the brain consumes a high fraction of total oxygen generating large amounts of reactive oxygen species [1,2]. Although brain antioxidant defenses function properly during most of human life, a number of neurodegenerative processes which involve redox-active iron accumulation become evident with age [3-5]. Iron is a pro-oxidant that in the reductive intracellular environment catalyses hydroxyl radical formation through the Fenton reaction [6]. At present, the crucial components of the iron homeostasis machinery have been identified. Thus, current efforts should be directed to the understanding of the mechanisms that regulate cellular iron levels and antioxidant defenses. This is of primary importance for the development of strategies to ameliorate iron accumulation and oxidative damage in neurons.

In vertebrates, cellular iron levels are post-transcriptionally controlled by the activity of iron regulatory proteins (IRP1 and IRP2), cytosolic proteins that bind to structural elements called iron-responsive elements (IREs). IREs are found in the untranslated region of the mRNAs of the major proteins that regulate cellular iron homeostasis: the transferrin receptor, involved in plasma-to-cell iron transport, and the iron-storage protein ferritin. IRP2-/- mice are born normal but in adulthood develop a movement disorder characterized by ataxia, bradykinesia and tremor [7]. IRP1-/- mice are normal with slight misregulation of iron metabolism in the kidney and brown fat [8]. Thus, IRP2 seems to dominate the physiological regulation of iron metabolism whereas IRP1 seems to predominate in pathophysiological conditions.

Iron is internalized into cells by the import transporter DMT1. Four DMT1 isoforms have been identified that differ in both the N-and the C-termini [9]. Two of the isoforms have a 3' iron responsive element (IRE) in their mRNA. Additional variation is given by exons 1A and 1B in the 5' end. Expression of DMT1 in response to iron availability follows a pattern similar to transferrin receptor [10], but its control by the IRE/IRP system is not clear [for review see [11]].

A new iron transporter, IREG1, also known as ferroportin or MTP1, was recently described [12,13]. The protein is expressed mainly in enterocytes and macrophages [reviewed in [14]]. In enterocytes IREG1 is responsible for iron efflux during the process of intestinal iron absorption, while in Kupffer cells IREG1 mediates iron export for reutilization by the bone marrow [15]. The presence of both DMT1 and IREG1 has been described in neurons, glioma cells and astrocytes [16-18]. The presence of IREG1 in neurons opens the possibility that they may be able to down-regulate intracellular iron concentration through its expression.

In this study we examined iron homeostasis in SH-SY5Y neuroblastoma cells and hippocampal neurons. We found that iron accumulation killed a large proportion of cells, but a sub-population became resistant to iron accumulation developing an adaptative mechanism intended to decrease intracellular iron content.

## Results

### Iron accumulation and cell death

**Figure 1:**
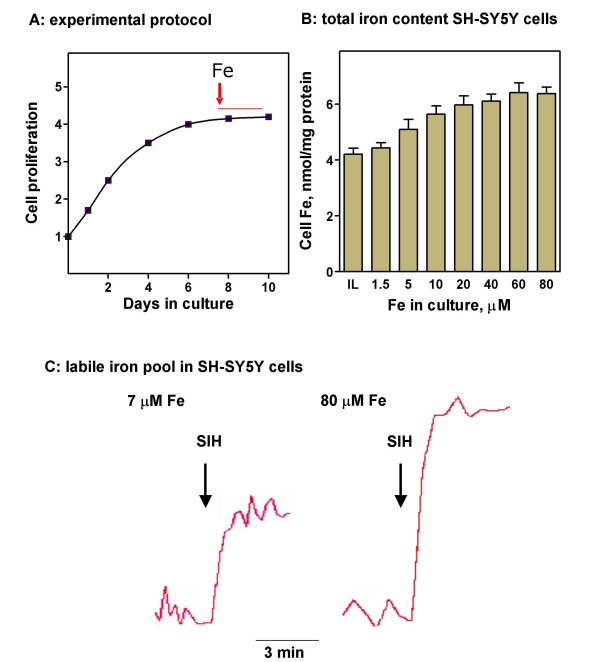
**Total and labile iron in the progressive iron accumulation model *A: ***SH-SY5Y cells were grown for 8 days and then challenged for 2 days with different concentrations of iron (range 1.5–80 μM). ***B: ***total iron content determined by mass spectrometry. Increased level of iron in the culture media produced increased levels of intracellular iron. Data is mean ± SD of 4 independent determinations. **C: **The calcein-sensitive iron pool increased at 80 μM Fe compared with 7 μM Fe. Representative tracings are shown.

**Figure 2:**
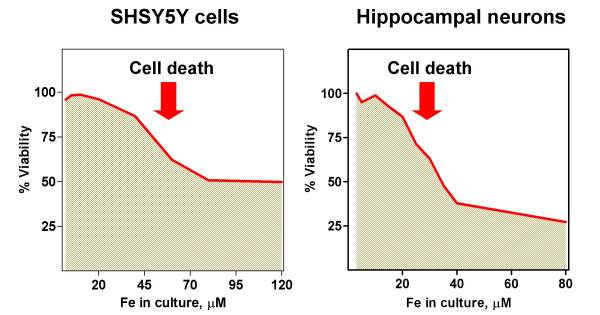
**Cell viability upon iron load. **SH-SY5Y cells and hippocampal neurons were subjected to the progressive iron accumulation protocol described in Figure 1A. Viability in the cell cultures was determined by the MTT method. Representative curves from 4 (SH-SY5Y cells) or 3 (hippocampal cells) independent experiments are shown.

Iron accumulation was determined in SH-SY5Y cells grown to confluence and then cultured for two days in media containing from 1.5 to 80 μM iron (Figure 1A). Total cell iron increased with increasing extracellular iron, reaching a plateau at 40–80 μM Fe (Figure 1B). The observed increase in cell iron was accompanied by increases in the labile iron pool (Figure 1C). Iron accumulation indeed caused loss of cell viability, with hippocampal neurons demonstrating higher sensitivity than SH-SY5Y cells to iron treatment (Figure 2). Nevertheless, a sub-population of cells survived to high iron concentrations. It was of interest to inquire into the processes underlying this adaptation, since they could help to understand iron accumulation observed in a number of neurodegenerative diseases. Consequently, we characterized the components of the iron homeostasis machine during the process of iron accumulation.

### Ferritin and DMT1 regulation

**Figure 3:**
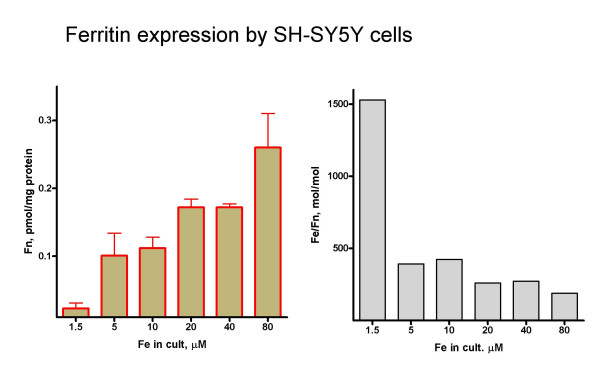
**Ferritin content *A: ***ferritin was determined in extracts from cells cultured for 2 days with different iron concentrations as described in Figure 1A. Data is mean ± SD from 4 independent determinations. ***B: ***iron : ferritin molar ratio. Total iron and ferritin total data were from Figure 1B and 3A, respectively. Ferritin synthesis response was larger that iron accumulation, an indication of a very active IRE/IRP system.

Ferritin, the main iron-storage protein in mammalian cells, is considered the first line of defense against iron overload. Increasing iron from 1.5 to 5 μM produced a robust 4-fold increase in cell ferritin content (Figure 3A). Further increases in iron induced additional increases in ferritin. At 80 μM extracellular iron, ferritin increased 11-fold compared to the basal 1.5 μM iron condition. In molar base, ferritin increased more than iron. The iron to ferritin mol : mol ratio decreased from 1500 at 1.5 μM Fe to 400 at 10 μM Fe to 200 at 80 μM Fe (Figure 3B).

**Figure 4:**
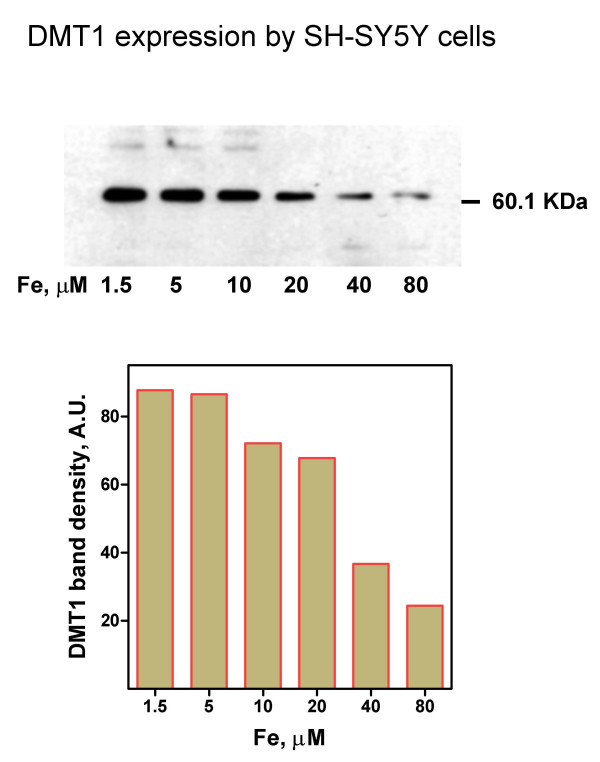
**Changes in DMT1 expression *Upper panel: ***DMT1 from cells cultured for 2 days with different iron concentrations was determined by Western blot. A continuous decrease in DMT1 expression was observed in the 1.5–80 μM Fe range. Shown is one of three similar experiments. ***Lower panel: ***densitometric analysis of the DMT1 bands shown in Figure 4A.

We further characterized iron homeostasis in SH-SY5Y cells by examining the expression of the iron importer DMT1 (Figure 4). A 3.5-fold decrease in DMT1 protein expression was observed when iron increased from 1.5 to 80 μM. The presence of DMT1 even at high iron concentration explains the sustained iron uptake observed at 80 μM Fe [19]. Thus, DMT1 activity persisted even under conditions of iron accumulation that preceded cell death.

### IREG1 expression and functionality

**Figure 5:**
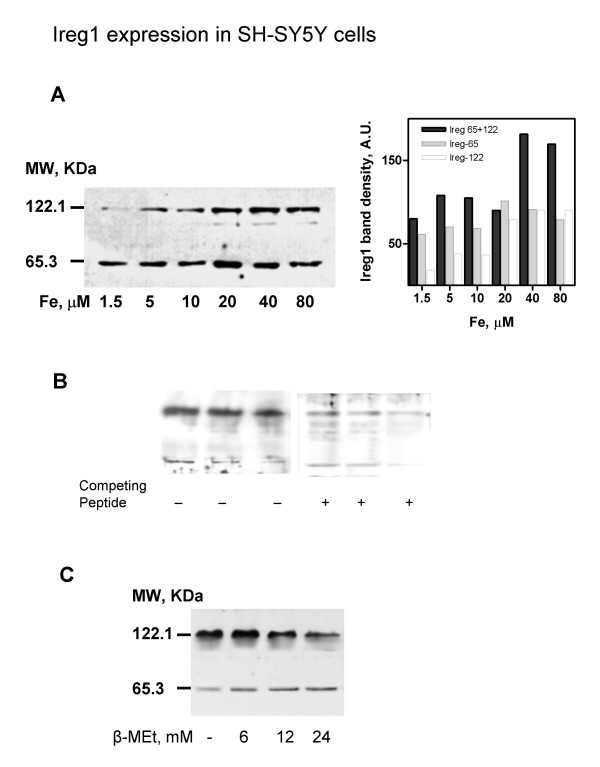
IREG1 expression increases upon increasing iron exposure. IREG1 from cells cultured for 2 days with different iron concentrations was determined by Western blot. A shows results of one of two similar experiments. Bands of 65.3 and 122.1 KDa were evident. The side panel shows the densitometric analysis of the bands. The 122.1 KDa band increased in the 1.5 to 40 mM Fe range. B: Both the upper, 122.1 KDa, band and the lower, 65.3 KDa, band diminished when the antibody was pre-incubated with peptide CGPDEKEVTKENQPNTSVV, prior to Western blot of 30, 20 and 10 mg of cell extract. C: treatment of 80 mM Fe extracts with increasing b-mercaptoethanol. A decrease in the 122.1 KDa band with increased b-mercaptoethanol was apparent.

Given that the presence of IREG1 in the central nervous system has been   reported [[16]], it was of interest to examine if it participates in neuronal iron homeostasis. Western blot analysis revealed that SH-SY5Y cells   expressed anti-IREG1 reactive bands of 65.3 and 122.1 KDa molecular weight (Figure 5A). Densitometric analysis revealed a 10-fold increase in the 122.1 KDa band in the 1.5 to 80 mM Fe range while the 65.3 band had a minor increase (Figure 5A). Both bands were eliminated if the antibody was   incubated with the immunogenic peptide before the assay (Figure 5B). A similar pattern was obtained with an independent anti-IREG1 antibody (the kind gift of Dr. David Haile). Thus, it is most likely that the 65.3 and   122.1 KDa bands correspond to the monomer and dimer of IREG1. The stability of the 122.1 KDa band was dependent of the concentration of b-mercaptoethanol in the sample buffer since increasing b-mercaptoethanol produced a shift in the 122.1 KDa /65.3 KDa band ratios (Figure 5C). It is possible that in neuronal cells Ireg1 tends to form S-S bridged dimers resistant to the electrophoresis conditions.

**Figure 6:**
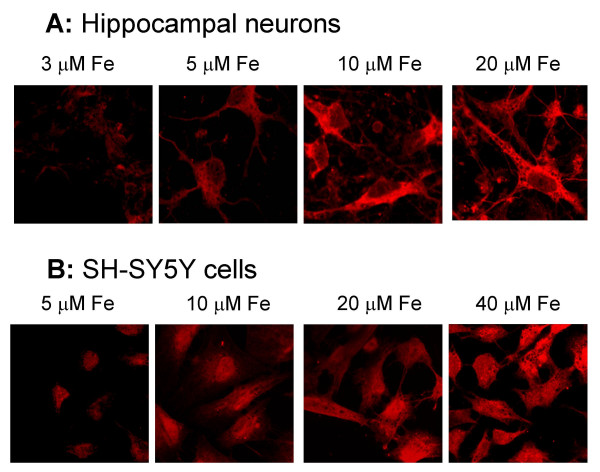
**Immunocytochemistry determination of IREG1. **Hippocampal neurons and SH-SY5Y cells, labeled with rabbit anti-IREG1 antibody and with Alexa-546-conjugated goat anti-rabbit IgG, were imaged in a confocal microscope. Shown are representative fields of cells cultured at different iron concentrations. Note the preferentially cytosolic distribution of IREG1.

The presence of IREG1 in SH-SY5Y neuroblastoma cells and hippocampal neurons was further documented by immunocytochemistry. IREG1 was detected in both types of cells, with a predominantly cytosolic distribution pattern (Figure 6). The levels of IREG1 expression were directly proportional to the amount of iron in the culture. Thus, it was determined by two independent methods that IREG1 expression in neuronal cells increased with cell iron content.

**Figure 7:**
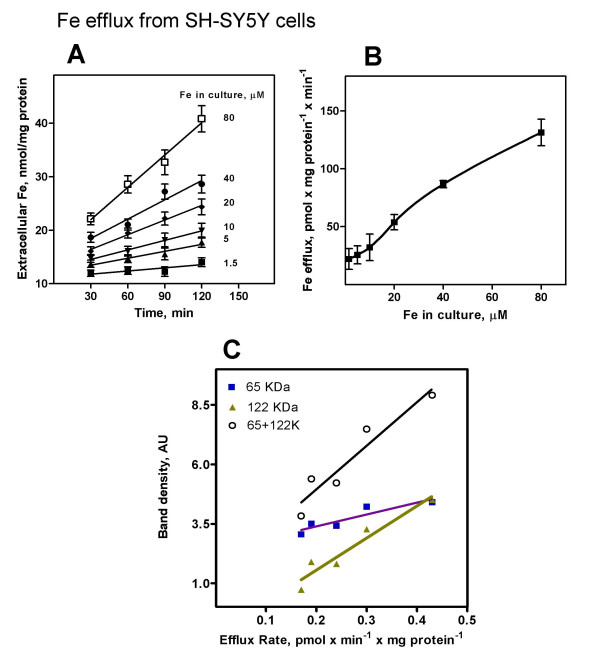
**Kinetics of iron efflux from SH-SY5Y cells. **SH-SY5Y cells were cultured for 2 days with varied amounts of iron as described in Figure 1A. The culture medium was changed to fresh DMEM and the kinetic of iron exit from the cells was followed by determining the concentration of iron in the medium by absorption spectrometry (***A***). Data is means ± SD from 3 experiments. The efflux rate, obtained from the slope of the curves in Figure 7A, was plotted against iron concentration during the 2-day culture period (***B***). In ***C***, the efflux rate was plotted against the density of the 65.3 KDa or 122.1 KDa bands. To that end, the 65.3 KDa or 122.1 KDa bands shown in Figure 5 were quantified by densitometry and paired with the efflux rates determined in Figure 7A at the same iron concentrations. A good correlation between efflux rate and the presence of the 122.1 KDa band was observed.

Efflux of iron from neurons has never been reported. In view of the presence of IREG1, we tested whether SH-SY5Y cells actually had an iron efflux function. To that end, iron efflux from cells pre-cultured for 2 days with varied iron concentrations was determined by atomic absorption spectrometry. This method was preferred to the use of radioisotopic iron since the latter could underestimate a putative iron efflux because of isotope dilution with the pre-existing iron pool (see Figure 1B). SH-SY5Y cells had discrete but measurable iron efflux activity (Figure 7A). The iron efflux rate increased markedly in the 20–80 μM Fe range (Figure 7B). Interestingly, the efflux rate correlated closely with the presence of the 122.1 KDa band, while the correlation between efflux activity and the 62.5 KDa band was weaker (Figure 7C).

## Discussion

The number of neurological diseases associated with iron accumulation in the brain underlines the need for increased knowledge of the mechanisms of brain iron homeostasis. In this study we show that iron accumulation by SH-SY5Y neuroblastoma cells and hippocampal neurons resulted in cell death of part of the population, while another fraction survived by adapting the expression of iron homeostasis proteins.

Iron content increased significantly as a function of Fe in the culture up to 20–40 μM Fe, increasing very little thereafter up to 80 μM Fe. Cell iron increase was accompanied by increased ferritin content. The increase in ferritin more than compensated for the increase in iron. Iron to ferritin mol ratios of 1500, 260 and 190 were obtained for 1.5, 20 and 80 μM Fe in the culture media. Thus, the IRE/IRP system of SH-SY5Y cells over-responded to iron accumulation in terms of ferritin expression. Despite the increase in ferritin, the LIP increased between 1.5 and 80 μM Fe. This finding clearly indicates that in SH-SY5Y cells the level of labile iron is a function of total iron, even in the presence of ample ferritin supply. It is possible that ferritin-stored iron contributes to the LIP each time that ferritin undergo lisosomal degradation.

Iron accumulation was accompanied by a marked decrease in DMT1 expression. Nevertheless, some DMT1 persisted even at 40–80 μM iron. The persistence of DMT1 at high iron concentrations could underline the continuous iron uptake observed under these conditions [19]. This is curious because at 40–80 μM Fe cells were dying. Sustained DMT1 expression points to the inability of neuronal cells to shut-off iron uptake and the need for additional defense mechanisms to prevent iron-mediated cell death.

The discovery of increased IREG1 expression in response to cell iron accumulation is a major break-through in the understanding of cell survival under conditions of iron accumulation. Total IREG1, and especially a putative IREG1 dimer, increased markedly in the 20–80 μM Fe range. Thus, in SH-SY5Y cells IREG1 is up-regulated by increased cell iron. Expressed IREG1 was functional since it associated with increased iron efflux activity. Iron efflux activity in astrocytes [18] and neurons (this work) indicate that iron efflux from brain cells is a dynamic process, and highlights the importance of iron transporters as determinants of iron accumulation.

The regulation of IREG1 expression is unknown but seems to be cell-specific. In enterocytes, IREG1 expression is induced by iron deficiency [13] while in macrophages iron increases IREG1 expression [20]. The findings reported here indicate that in neuronal cells IREG1 has a macrophage-like regulation. This is certainly the case for cells in the 40–80 μM range that survived to iron accumulation. IREG1's predominantly cytosolic distribution pattern is similar to that of Kupffer cells [12]. Again, this distribution points to macrophage-like behavior of neuronal IREG1. In examining brain biopsies from Alzheimer's patients an intriguing question arises: Why do some neurons die or present evident signs of degeneration while others in the vicinity show a normal phenotype? Extrapolating on the data presented here, it is tempting to hypothesize that surviving neurons induce IREG1 expression while sick neurons do not. Nevertheless, at present we cannot exclude that other regulatory molecules may play a pivotal role under these conditions.

## Conclusions

Hippocampal neurons and SH-SY5Y cells displayed an active system to regulate iron content. Nevertheless, this system was unable to block iron accumulation which resulted in death of part of the cell population. Another fraction of the cell population developed an adaptative mechanism that includes decreased expression of the import transporter DMT1 and increased expression of ferritin and the efflux transporter IREG1. The finding that neurons regulate the expression of functional IREG1 opens new avenues for the understanding and possible treatment of iron-related neurodegenerative processes.

## Methods

### Antibodies and immunodetection

Antibody D-1, prepared against the C-terminal end of the IRE-containing isoform of DMT1 was used as described previously [10]. Additionally, a rabbit polyclonal antibody against peptide CGPDEKEVTKENQPNTSVV, corresponding to the consensus sequence of human, rat and mouse carboxyl-terminal sequence of IREG1, was obtained from BioSonda, Chile .

### Western analysis

Cell extracts, cells were prepared treating cells with lysis buffer (50 μl per 1 × 106 cells of 10 mM MOPS, pH 7.5, 3 mM MgCl2, 40 mM KCl, 1 mM phenylmethylsulfonyl fluoride, 10 μg/ml leupeptin, 0.5 μg/ml aprotinin, 0.7 μg/ml pepstatin A, 5% glycerol, 1 mM dithiothreitol, 0.1% Triton X-100). The mixture was incubated for 15 min on ice and centrifuged for 10 min at 5,000 × g. Protein concentrations were determined using the bicinchoninic acid (BCA) protein assay. The supernatant was stored at -70°C. For Western analysis, 30 micrograms of protein from each sample were boiled in Laemmli sample buffer for 5 min and subjected to SDS-PAGE on a 7.5% acrylamide gel. Proteins were transferred to nitrocellulose membrane and blocked for 1 hr at 25°C with 5% nonfat dry milk in blocking saline (20 mM Tris, 0.5 M NaCl, 0.05% Tween-20). Membranes were incubated with primary antibody overnight at 4°C, rinsed with blocking saline and incubated with horseradish peroxidase-conjugated anti-rabbit IgG antibody for 1 hr at 25°C. Transferred proteins were detected with a peroxidase-based chemiluminiscence assay kit (SuperSignal, Pierce Chem. Co., Rockford, IL). Chemiluminiscence was detected using a Molecular Imager FX device (Bio-Rad, Hercules, CA). The bands were quantified by densitometry using the Quantity One (Bio-Rad) software.

### Cell culture and iron challenge

Human neuroblastoma SH-SY5Y cells (CRL-2266, American Type Culture Collection Rockville, MD), were seeded at 1 × 105 cells in 2-cm^2 ^plastic wells and cultured in a 5 % CO_2 _incubator in MEM/F12 medium supplemented with 10 % fetal bovine serum and 5 mM glutamine. The medium was replaced every two days. Under these conditions, doubling time was about 48 hours. After 8 days in culture, the culture reached a steady-state number of cells. At this time, cells were challenged with iron for the next two days as described [19]. In brief, low-iron culture media was supplemented with either 1, 5, 10, 20, 40 or 80 μM Fe^3+ ^as the complex FeCl_3_-sodium nitrilotriacetate. Cell viability was quantified by the MTT assay (Molecular Probes, OR) following the manufacturer's instructions. This model of iron loading attempts to replicate neuronal iron accumulation that occurs during life [4].

Hippocampal neurons were prepared from E18.5 rat embryos [21]. Neurons were plated over poly-L-lysine coated cover slips at 100,000 cells/cm^2^. Cultures were maintained in 10% bovine serum until 3 hours after plating, when the culture medium was replaced with medium containing B27 supplement [22]. After 3 days in culture, the cells were challenged with iron as described above.

### Labile iron pool

The intracellular labile or reactive iron pool of neuroblastoma cells was determined as described [23,24]. The increase in fluorescence after the addition of SIH chelator is directly proportional to the iron labile pool, i.e., iron in complexes with affinity constant < 10^6^.

### Immunocytochemistry

Cells grown in cover slips were sequentially fixed with 2% and 4% parafolmaldehyde (PFA) in Eagles' MEM, and then washed three times with phosphate-buffered saline (PBS). The fixed cells were permeabilized with Triton-X-100 (0.2%) in PBS at room temperature for 3 min and blocked with defatted milk (10%) in PBS for more than 1 h. The cells were incubated with anti-IREG1 antibody (1:500) overnight at 4°C, washed with PBS and then incubated with Alexa-546-conjugated goat anti-rabbit IgG. The labeled cells were observed with a Zeiss LSM 510 Meta confocal laser scanning microscope.

### Data analysis

Variables were tested in triplicates, and experiments were repeated at least twice. Variability among experiments was <20%. One-way ANOVA was used to test differences in mean values, and Turkey's post-hoc test was used for comparisons (In Stat program from GraphPad Prism). Differences were considered significant if P < 0.05.

## Authors' contributions

MTN conceived of the study, participated in its design and coordination and drafted the manuscript. PA performed the experiments with hippocampal neurons, did the ferritin assays and participated in the analysis and interpretation of data. MN optimized the immunocytochemistry detection of IREG1, performed the confocal microscope observations and participated in the analysis and interpretation of data. VT did the Western blot, labile iron pool and viability assays and contributed to the discussion of the results. MA set up the method to determine total Fe concentration, did the sample and control measures of iron and participated in the analysis and interpretation of data. All coauthors participated in refining the text.
